# Biophysical, thermo‐physiological and perceptual determinants of cool‐seeking behaviour during exercise in younger and older women

**DOI:** 10.1113/EP091533

**Published:** 2023-11-16

**Authors:** Alessandro Valenza, Hannah Blount, Antonino Bianco, Peter R. Worsley, Davide Filingeri

**Affiliations:** ^1^ ThermosenseLab, Skin Sensing Research Group, School of Health Sciences The University of Southampton Southampton UK; ^2^ Sport and Exercise Sciences Research Unit, SPPEFF Department University of Palermo Palermo Italy; ^3^ PRESSURELAB, Skin Sensing Research Group, School of Health Sciences The University of Southampton Southampton UK

**Keywords:** biophysical cues, exercise, perceptual responses, thermal behaviours, women

## Abstract

Women continue to be under‐represented in thermoregulatory research despite their undergoing unique physiological changes across the lifespan. This study investigated the biophysical, thermo‐physiological, and perceptual determinants of cool‐seeking behaviour during exercise in younger and older women. Eleven younger (25 ± 5 years; 1.7 ± 0.1 m; 63.1 ± 5.2 kg) and 11 older women (53 ± 6 years; 1.7 ± 0.1 m; 65.4 ± 13.9 kg) performed a 40‐min incremental cycling test in a thermoneutral environment (22 ± 1.7°C; 36 ± 4% relative humidity). Throughout the test, participants freely adjusted the temperature of a cooling probe applied to their wrists to offset their thermal discomfort. We continuously recorded the probe–wrist interface temperature to quantify participants’ cool‐seeking behaviour. We also measured changes in participants’ rate of metabolic heat production, core and mean skin temperatures, and skin wetness. Finally, we body‐mapped participants’ skin heat, cold and wetness sensitivity. Our results indicated that: (1) older and younger women exhibited similar onset and magnitude of cool‐seeking behaviour, despite older women presented reduced autonomic heat‐dissipation responses (i.e., whole‐body sweat losses); (2) older women's thermal behaviour was less determined by changes in core temperature (this being a key driver in younger women), and more by changes in multiple thermo‐physiological and biophysical parameters (i.e., physical skin wetness, temperature and heat production); (3) older women did not present lower regional skin thermal and wetness sensitivity than younger women. We conclude that predictions of female cool‐seeking behaviours based on thermo‐physiological variables should consider the effects of ageing. These findings are relevant for the design of wearable cooling systems and sports garments that meet the thermal needs of women across the lifespan.

## Abstract (highlights)

- **What is the central question of this study?**
  How does cool‐seeking behaviour during exercise and its underlying biophysical, thermo‐physiological and perceptual determinants change with ageing in women?
- **What is the main finding and its importance?**
  Younger and older women exhibited similar onset and magnitude of cool‐seeking behaviour, despite older women presenting reduced autonomic heat‐dissipation responses. Furthermore, older women's thermal behaviour was less determined by changes in core temperature, and more by changes in multiple thermo‐physiological and biophysical parameters. These findings are relevant for the design of wearable cooling systems and sports garments that meet the thermal needs of women across the lifespan.

## 1 INTRODUCTION

Hot weather and heat extremes have severe detrimental effects on individuals’ health, comfort and productivity, both at rest and during physical activity (Ebi et al., 2021; Vargas et al., 2023). Behavioural thermoregulation, that is, the ability to detect the thermal state of one's own body and surroundings and to actively pursue thermal comfort and homeostasis, represents humans’ first line of defence from the heat (Schlader & Vargas, 2019). By expanding our ability to withstand life‐threatening thermal stress (e.g., consider how seeking physical cooling supports heat‐stress tolerance), thermal behaviours effectively complement autonomic thermoregulatory responses (i.e., vasomotion and sweating) in the regulation of body temperature during exercise and heat stress (Cramer & Jay, 2019).

Physiological and perceptual signals from the body play an important role in the behavioural drive to maintain thermal homeostasis (Vargas et al., 2020). For example, when exposed to heat during rest or light activity, thermoreceptors in the skin and body core drive thermal discomfort and initiate cooling behaviours; this response is also augmented by the physical build‐up of sweat on the skin (i.e., physical skin wetness) during more intense physical activity (Vargas et al., 2023). However, we know that physiological (e.g., thermo‐effector) and perceptual sensitivity (i.e., central and peripheral temperature and wetness sensing) to thermal stress may vary greatly at an individual level, due to a complex interaction amongst morphological (e.g., body mass and surface area), demographical (e.g., sex and age) (Cramer & Jay, 2015), and neurophysiological factors (e.g., regional and age‐dependent changes in thermoreceptors density) (Typolt & Filingeri, 2020; Valenza et al., 2019). These individual differences may in turn modulate behavioural thermoregulation in groups differing by age or sex, such that, for example, older adults may have a delayed initiation of protective thermal behaviours to heat stress (Millyard et al., 2020). Yet, despite recent advances in our understanding of thermal behaviours (Schlader & Vargas, 2019), mechanistic research on the determinants of thermal behaviours and of their individual variability with sex and age remain limited (Vargas et al., 2023). Furthermore, biophysical factors and their individual variability have been rarely considered in studies of thermal behaviours, despite their relevance in driving individual variability in thermoregulatory responses during exercise (Cramer & Jay, 2015).

In the context of individual variability as a function of biological sex, recent evidence indicates that women may engage in thermoregulatory behaviours to a greater extent than men during exercise, despite exhibiting similar heat‐dissipating thermo‐effector responses (Vargas et al., 2019a). This is presumably due to different requirements for thermal comfort between the sexes, secondary to sex differences in the management of energy expenditure arising from thermoregulatory effector responses (Vargas et al., 2019a), as considered below. From a physiological standpoint, we know that during exercise, the decision to behaviourally thermoregulate seems to be preceded by modest changes in skin blood flow, which occur prior to the activation of more profound increases in skin blood flow and sweating during heat stress (Schlader & Vargas, 2019). This arrangement may be physiologically beneficial given that small changes in skin blood flow and thermal behaviour have a relatively lower physiological cost than more energy demanding autonomic thermo‐effectors such as sweating (Schlader et al., 2018) (although it should be noted that the initiation of thermal behaviour requires a certain level of subjective thermal discomfort (Sedilla & Maeda, 2022), which is likely driven by warm and (sweat‐induced) wetness perceptions). In this context, if we consider sex‐based differences in thermo‐effector responses, that is, evaporative heat loss and local sweat rate, we know that women exhibit lower maximum sweating capacity than men only under conditions of exercise‐heat stress that has a very high requirement for evaporative heat loss (i.e., >∼300 W/m^2^; Gagnon & Kenny, 2012). However, these do not represent scenarios that individuals may engage with on a regular basis or for long periods of time (Gagnon & Kenny, 2012). Nevertheless, women have been observed to present lower sweat output per gland (Buono & Sjoholm, 1988) and greater sensitivity to skin wetness (Shapiro et al., 1980), such that, in conditions of high relative humidity, they may display a reduced sweat rate compared to their male counterparts. This reduction in sweat rate may be advantageous, as it would allow retention of body water in environmental conditions not conducive to evaporative heat dissipation (Greenfield et al., 2023). While this may be physiologically beneficial from a fluid retention perspective, it has little or nothing to do with heat dissipation (Wang et al., 2018). Considering the physiological evidence above on female‐specific autonomic thermo‐effector responses, it may therefore be reasonable to deduce that the more sensitive thermal behaviours observed in young females, including a tendency to seek cooler conditions or downregulate pace, may represent a unique adaptive mechanism to offset sex‐related differences in thermo‐effector responses (i.e., sweating) to the heat (Vargas et al., 2019a).

Aside from the physiological mechanisms that may underlie female‐specific thermal behaviours, it is well‐established that young women also differ from men in their thermal preferences (Wang et al., 2018), likely due to a heightened (perceptual) thermal sensitivity (Inoue et al., 2016), which cannot be entirely ascribed to morphological factors (Filingeri et al., 2018; Luo et al., 2020). Furthermore, young women often report greater thermal discomfort at the same absolute temperature when compared to men, and they frequently detect these thermal changes sooner (Frank et al., 1999; Hashiguchi et al., 2010; Luo et al., 2020; Valenza et al., 2019). Finally, we have recently reported that young women are more sensitive to skin wetness than males (Valenza et al., 2019). When combined with the presence of a lower maximum sweating capacity than that of men, a greater sensitivity to warmth and skin wetness in women could offer protective benefits in hot environments if it were to facilitate an earlier onset of thermal behaviours.

The physiological and perceptual considerations discussed above have important implications for understanding female‐specific thermoregulatory behaviours, thermal comfort and heat stress resilience. However, women continue to be largely unrepresented in autonomic and behavioural heat‐stress research (Hutchins et al., 2021). By way of an example, no mechanistic study has thus far investigated how thermal behaviours (and their underlying physiological and perceptual correlates) change with ageing in women. This is surprising when considering that women are a group of individuals that undergo unique morphological, physiological and hormonal changes across the lifespan. For example, consider the impact of the menstrual cycle, pregnancy and menopause, all of which are accompanied by both short‐ and long‐term effects on female body temperature regulation, heat tolerance and thermal comfort (Carter et al., 2023; Frank et al., 1999; Greenfield et al., 2023; Hashiguchi et al., 2010). Furthermore, we have recently observed in males that ageing reduces sensitivity to skin wetness (Wildgoose et al., 2021). This leads to the yet‐to‐be‐answered question of whether a similar loss of skin wetness sensitivity occurs in older women and whether this may in turn worsen behavioural thermoregulation in older women. Altogether, these knowledge gaps provide significant barriers to develop interventions (e.g., personalized cooling) and solutions (e.g., body‐mapped sport garments) that meet the thermal needs of women across different life stages, and that ultimately promote an active lifestyle at a time of climate change.

The aim of this study was to comprehensively investigate the biophysical, thermo‐physiological and perceptual determinants of thermal (cool‐seeking) behaviour during exercise in younger and older women. To do so, we used a unique combination of perceptual body mapping of temperature and wetness sensitivity, with state‐of‐the‐art biophysical and thermo‐physiological measurements in younger and older women undergoing an ecologically valid behavioural paradigm during exercise. We hypothesized that (1) older women would present delayed and less effective cool‐seeking behaviours than their younger counterparts; (2) the relative contribution of biophysical, thermo‐physiological and perceptual parameters to cool‐seeking behaviour would change with ageing due to age‐related declines in thermo‐physiological and perceptual functions; and (3) regional skin wetness sensitivity would be significantly lower in older women, as recently observed in the case of aged men.

## 2 METHODS

### 2.1 Ethical approval

The testing procedures were explained to each participant, and they all gave written informed consent for participation. The study was approved by the Research Integrity and Governance team of University of Southampton (ERGOII 72799). All testing procedures were in accordance with the tenets of the *Declaration of Helsinki* (note: the study was not registered in a database). All testing took place at Southampton (UK) between December 2022 and January 2023.

### 2.2 Participants

We used a convenience sampling approach and recruited 11 younger (25 ± 5 years; 1.68 ± 0.07 m; 63.1 ± 5.2 kg) and 11 older (53 ± 6 years; 1.67 ± 0.06 m; 65.4 ± 13.9 kg) non‐smoking, recreationally active (i.e., >3 exercise sessions per week) female participants, with no history of cardiovascular, neurological and skin‐related conditions (e.g., eczema) from the population of Southampton and Southampton University to take part in the study.

Participants' characteristics are presented in Table 1. Participants were matched for body surface area (BSA), which resulted in the same proportion of their body being stimulated by the thermal probes that we used both to deliver thermal and wet stimuli at rest (see ‘Body mapping experiment’, section 2.3.1) as well as to evaluate thermal behaviours during physical activity (see ‘Thermal behaviour experiment’). Regarding their physical fitness, we did not purposely match groups for their maximum aerobic fitness (i.e., extrapolated ${\ddot{V}}_{O_{2}\max}$). However, extrapolation of ${\ddot{V}}_{O_{2}\max}$ from the indirect calorimetry data collected during the submaximal cycling tests (see ‘Thermal behaviour experiment’) indicated that younger and older women had similar maximal aerobic fitness (Table 1).

**TABLE 1:** Participants’ characteristics.

Characteristics	Younger women (*n* = 11)	Older women (*n* = 11)	*P*
Age (years)	24.9 ± 4.9	52.9 ± 6.2	<0.0001^*^
Height (m)	1.68 ± 0.07	1.67 ± 0.06	0.809
Weight (kg)	63.1 ± 5.2	65.4 ± 13.3	0.597
BSA (m^2^)	1.71 ± 0.09	1.74 ± 0.19	0.701
BMI (kg/m^2^)	22.47 ± 2.29	23.49 ± 4.27	0.482
${\ddot{V}}_{O_{2}\max}$ (predicted) (ml/kg/min)	34.2 ± 9.19	35.08 ± 9.86	0.831

*Note*: Values are reported as means ± SD. ^*^Statistically significantly different (*P* < 0.05).

We did not control for the menstrual phase based on preliminary evidence that both thermal sensation and exercise performance in females may not be independently modified by menstruation (Matsuda‐Nakamura et al., 2015; McNulty et al., 2020). Nevertheless, we collected participants’ self‐reports of the corresponding day of the menstrual cycle they were in at the time of testing. Younger participants self‐reported being spread across a typical 28‐day menstrual cycle (day of cycle: 20 ± 12), with two of them reporting irregular periods, and three of them taking oral contraceptives. Regarding the older participants, four of them self‐reported having regular periods (day of cycle: 20 ± 6); the remaining seven participants self‐reported to be menopausal (i.e., no longer having regular periods for at least 6 months). Among the seven menopausal participants, four of them reported being under hormone replacement therapy and one of them to be taking hormonal contraception. Aside from those participants (*n* = 7) reporting taking oral contraceptives (*n* = 4) or being under hormone replacement therapy (*n* = 3), none of the remaining participants (*n* = 15) reported taking any medication at the time of testing.

Participants were instructed to refrain from (1) performing strenuous exercise in the 48 h preceding testing; (2) consuming caffeine or alcohol in the 24 h preceding testing; and (3) consuming food in the 3 h preceding testing.

### 2.3 Experimental design

All participants took part in one testing session, during which two separate experiments were performed: (1) body mapping of skin thermal and wetness sensitivity, and (2) thermal behaviour during exercise. The aim of the first experiment was to determine regional patterns of skin sensitivity to temperature and wetness at rest, and their potential differences between age groups. Temperature and wetness sensing are implicated in thermal behaviours (Vargas et al., 2020); hence this first experiment was conceived to evaluate how any age‐dependent change in temperature and wetness sensing across the body may have subsequently contributed to differences in cool‐seeking behaviour (i.e., the perceptual determinant). The aim of the second experiment was to test cool‐seeking behaviour during exercise‐heat stress and to determine its biophysical and thermo‐physiological determinants in both age groups. Altogether, experiments 1 and 2 provided a comprehensive analysis of the perceptual, biophysical and thermo‐physiological determinants of cool‐seeking behaviours in younger and older women. The sections below provide methodological details for each experiment.

#### 2.3.1 Body mapping experiment

We used a single‐blind psychophysical approach based on a well‐established quantitative sensory test of skin wetness sensing that we have developed (Filingeri et al., 2014a) to map differences in regional thermal and wetness sensitivity at rest in a thermoneutral environment (ambient temperature: 22.0 ± 1.7°C; relative humidity: 36 ± 4%).

The quantitative sensory test consisted of participants having to report the perceived magnitude of local thermal and wetness perceptions arising from the short‐duration (i.e., 5 s) static application of a cold‐wet (i.e., 5°C below local skin temperature (*T*_sk_)), neutral‐wet (i.e., temperature equal to local *T*_sk_), and warm‐wet (i.e., 5°C above local *T*_sk_) hand‐held temperature‐controllable probe (NTE‐2A; Physitemp Instruments LLC, Clifton, NJ, USA; surface area: 1.32 cm^2^; water content: 0.8 mL). Participants reported the magnitude of their local perceptions on two digital visual analogue scales for thermal sensation (length 200 mm; anchor points: 0, very cold; 100, neutral; 200, very hot) and wetness perception (length: 100 mm; anchor points: 0, dry; 100, completely wet). We used stimuli whose temperatures were relative to the local *T*_sk_ pre‐stimulation (i.e., ±5°C or equal to local *T*_sk_) to account for intra‐ and inter‐individual variability in local *T*_sk_, and to ensure that the same relative thermal stimulus would be applied to all participants (Darian‐Smith, 1984).

We mapped thermal and wetness sensitivity at four different locations over the body: the centre of the forehead (i.e., 5 cm above the pupillary line), the posterior neck (i.e., over the process spinous of cervical 4), the centre of the volar wrist (i.e., 4 cm above the carpal line), and the dorsal foot (i.e., midpoint between the second and third metatarsal joints). We chose those body regions because: (1) they present high exercise‐induced local sweat rates (e.g., forehead) (Smith & Havenith, 2012); (2) they are reported among the most thermally sensitive areas (e.g., neck and wrist) (Nakamura et al., 2013); and (3) they were recently reported to be more evidently impacted by ageing (e.g., foot) (Wildogoose et al., 2021).

As with previous studies (Filingeri et al., 2014a, 2014b, 2018), all participants were blinded to the nature and application of the stimuli to limit expectation biases, and they were only informed about the location of the stimulation. Furthermore, participants underwent a systematic familiarization and calibration to the testing procedures and perceptual scales prior to testing (Filingeri et al., 2014a, 2018). The same investigator performed all testing.

#### 2.3.2 Thermal behaviour during exercise

To evaluate age‐dependent changes in thermal behaviour during exercise, we used a cool‐seeking behaviour paradigm modified from that previously developed by Schlader et al. (2018) and Vargas et al. (2018a, 2018b, 2020, 2019a). Briefly, participants underwent a 40‐min incremental exercise test (i.e., the workload increased at 10‐min intervals from 20 to 80 W) on a semi‐recumbent cycle‐ergometer (Lode B.V., Groningen, The Netherlands) in a thermoneutral environment (ambient temperature: 22.0 ± 1.7°C; relative humidity: 36 ± 4%). This incremental exercise test was designed to induce meaningful increases in the rate of metabolic heat production, with subsequent rises in core temperature and physical, sweat‐induced skin wetness, both of which have been previously demonstrated to trigger cool‐seeking behaviours (Vargas et al., 2018a, 2018b, 2020, 2019a).

**FIGURE 1:**
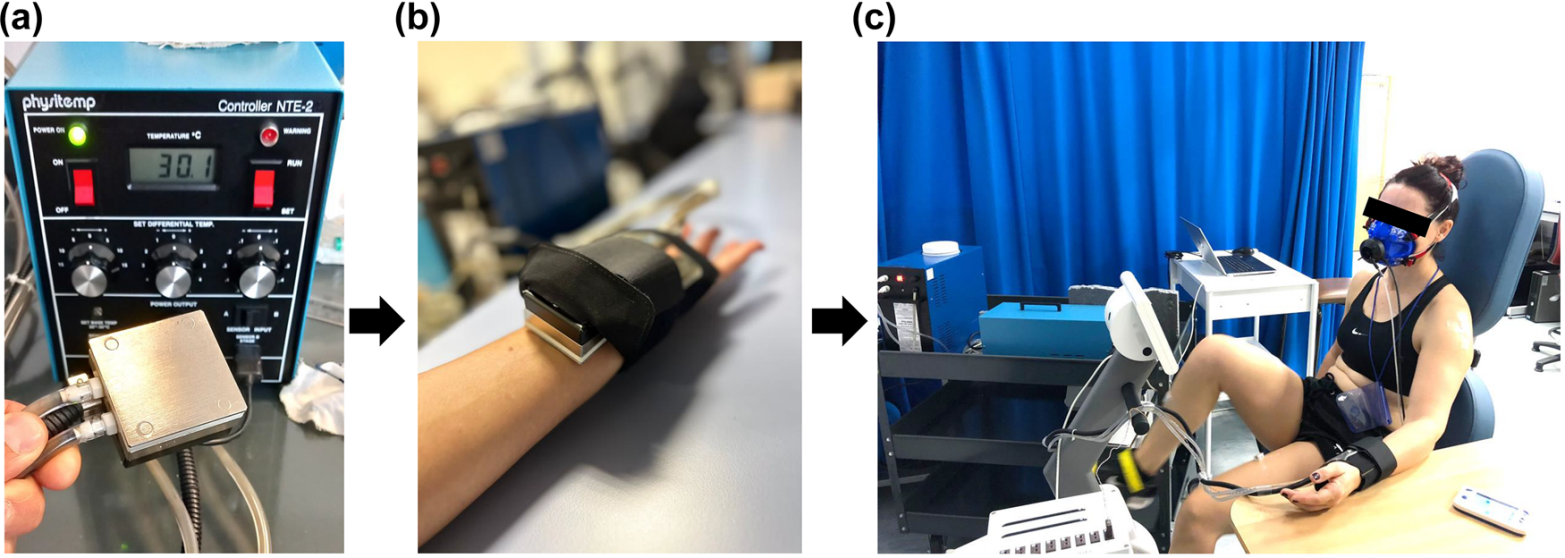
Schematic representation of the experimental set‐up, highlighting the square thermal probe and control panel used to assess cool‐seeking behaviour (a) via its application at the wrist (b) during the exercise test (c).

Throughout the exercise test, participants were equipped with a squared thermal probe (NTE‐2A; Physitemp Instruments; surface area: 25 cm^2^), which was placed on their left volar wrist and secured by means of a custom‐made arm band (Figure 1). The probe was connected to a control unit with three rotary knobs, which allowed control of the probe's temperature with switched steps of 5, 1 or 0.1°C. The baseline probe's temperature was determined individually based on the participant's wrist skin temperature (measured via an infrared camera), which ranged between 29.5 and 33.5°C. Participants were instructed to freely adjust the temperature of the thermal probe via the three rotary knobs during the exercise test to offset whole‐body thermal discomfort arising from exercise‐induced heat stress. Participants underwent familiarization and practice with the thermal probe before the experimental tests. Specifically, participants were allowed and encouraged to handle the probe and to assess how their operation of the three rotary knobs (switched steps of 5, 1 or 0.1°C) resulted in perceivable changes to the probe's surface temperature. Participants were allowed to operate the probe unit for however long was needed to confirm that they understood both the probe's operation and the actual protocol (i.e., they would freely use the thermal probe secured at their wrist as and when needed during the exercise trial to offset whole‐body thermal discomfort). Due to the ease of operation of the probe, we found that participants required ∼10 min to become fully familiar with the set‐up.

Probe–skin interface temperature was measured continuously (2 Hz) with a micro‐thermocouple (Omega Engineering, CT, USA) secured to the centre of the volar wrist. Changes in probe‐skin interface temperature, as driven by participants’ control of the thermal probe, provided an objective and continuous index of the onset and dynamic changes in cool‐seeking behaviour during exercise. We chose the volar wrist (as opposed to, e.g., the neck, a skin site extensively used in thermal behaviour studies by Vargas et al. (2018a, 2018b, 2020, 2019a) due to its accessibility and relevance for the development of wearable personal comfort systems (e.g., smart wrist bands (https://embrlabs.com; Zhang et al., 2015). Importantly, evidence indicates that a skin site may be equally as thermally sensitive as the neck to both cooling and heating (Nakamura et al., 2013), thereby providing an opportunity to compare the mechanisms underlying body region‐dependent cool‐seeking behaviours.

Throughout the exercise test, we also monitored continuously participants’ rate of metabolic heat production (*H*_prod_), gastro‐intestinal (core) temperature (*T*_core_), mean and local skin temperatures (*T*_sk_), and mean (physical) skin wetness (*w*) in both older and younger women. This was done to establish age‐related differences in the relative contribution of biophysical and thermo‐physiological parameters (and perceptual – see Section 2.3.1, ‘Body mapping experiment’) to cool‐seeking behaviour during exercise.

### 2.4 Experimental protocol

Participants arrived at the laboratory on testing days, having previously swallowed (i.e., 3 h prior to testing) a telemetric pill used for the measurement of *T*_core_ (BodyCAP, Hérouville Saint‐Claire, France), and underwent preliminary measurements and preparation.

First, we assessed participants’ semi‐nude body mass on a precision scale (Model 874; Seca GmbH, Hamburg, Germany) and their height on a wall stadiometer. From this point onward, participants were no longer allowed to drink water. Participants then changed into running shoes, shorts and sport‐bra. At this point, they underwent 20 min of resting on a chair to adjust to the environmental conditions. During this time, participants were familiarized with the experimental procedures of the body mapping experiment, including our well‐established perceptual calibration procedures (on a visual analogue scale), as detailed by Valenza et al. (2019). Upon termination of the calibration, the quantitative sensory test commenced and lasted 20 min. Briefly, we used a washable marker to mark the skin sites to be stimulated and depending on the body region to be tested, we first recorded the local *T*_sk_ of the testing site with an infrared camera (ER53, FLIR Systems, Wilsonville, OR, USA). We then determined the temperature of the first wet stimulus (e.g., cold wet, 5°C below local skin temperature) and applied a 100% cotton fabric on the hand‐held, round thermal probe (surface area: 1.32 cm^2^; NTE‐2A; Physitemp Instruments), which was then wetted with a pipette with 0.8 mL of water to ensure its full saturation. Following a verbal warning, the wet stimulus was applied statically on the participant's skin for 5 s, following which the participant was encouraged to rate her very first thermal and wetness perception. Application pressure was not measured but was controlled to be sufficient to ensure full contact with the skin region, at the same time not resulting in pronounced skin indention. Upon acquisition of the perceptual rating, we removed the stimulus, gently dried the skin, and then repeated the same procedure for the other stimuli (e.g., neutral and warm wet) on the same skin site, before proceeding to the next skin region. The order of testing regions (*n* = 4) and stimuli (e.g., warm vs. neutral vs. cold wet) was designed to minimize any order effect among participants (i.e., each of the 11 participants in each group underwent a different order of regions × stimuli combination).

Upon completion of the quantitative sensory test, participants were prepared and instrumented to commence the thermal behaviour experiment.

First, we taped four wireless thermistors (iButtons, Maxim, San Jose, USA) directly onto the skin of the chest, shoulder, thigh and shin to record local *T*_sk_ for the estimation of mean *T*_sk_ according to the following equation (Ramanathan, 1964):

$$\begin{array}{ccc} \mathrm{Mean}T_{\mathrm{sk}} & = & \left(\mathrm{left}\mathrm{upper}\mathrm{chest}T_{\mathrm{sk}}\times 0.30\right)\\ & & +\left(\mathrm{left}\mathrm{front}\mathrm{shoulder}T_{\mathrm{sk}}\times 0.30\right)\\ & & +\left(\mathrm{right}\mathrm{anterior}\mathrm{thigh}T_{\mathrm{sk}}\times 0.20\right)\\ & & +\left(\mathrm{right}\mathrm{shin}T_{\mathrm{sk}}\times 0.20\right) \end{array}$$

Furthermore, we placed four additional temperature and humidity sensors (Hygrochron, iButtons, Maxim) to the contralateral chest, shoulder, thigh and shin skin sites, to record local skin temperature and relative humidity to be used for the estimation of physical local (*w*_local_) and mean skin wetness (*w*). These sensors were placed on 3D‐printed cases that raised the sensor 6 mm off the skin, while ensuring airflow around the skin site. The distance of 6 mm was chosen to minimize artificial supersaturation of the sensor due to direct contact with sweat secreted onto the skin (Vargas et al., 2018a, 2019b). Local relative humidity and skin temperature were then used to determine the water vapour pressure of the skin using standard calculations as previously reported (Filingeri et al., 2015). *w*_local_ was calculated according to the methods of Gagge (1937), that is, as the ratio between the evaporative heat flux gradient between the humidity at the skin and in the air, and the maximal evaporative heat flux gradient for a totally wet skin (Filingeri et al., 2015; Gagge, 1937). Mean *w* was calculated as the equally weighted average of all four local skin wetness sites (Vargas et al., 2018a).

At this point, participants mounted the semi‐recumbent cycle‐ergometer, and they commenced an extensive familiarization with the thermal probe used for the cool‐seeking behaviour evaluation. Upon completion of the familiarization, participants confirmed understanding of the protocol, that is, they would freely use the thermal probe secured at their wrist as and when needed during the exercise trial to offset whole‐body thermal discomfort. Finally, participants were instrumented with a face mask connected to a breath‐by‐breath gas analyser (Quark CPET Metabolic Cart, Cosmed, Rome, Italy), which was used throughout the exercise trial to estimate the rate of metabolic heat production via partitional calorimetry, as extensively described by Cramer & Jay (2019).

Participants were then instructed to start cycling at an initial workload of 20 W with a comfortable, self‐selected cadence (note, the ergometer was set on a hyperbolic mode to maintain workload intensity independently of cycling cadence). This workload intensity was increased by 20 W every 10 min, until the maximum exercise duration was reached (i.e., 40 min and 80 W). While we acknowledge that prescribing exercise intensity at a fixed rate of metabolic heat production is relevant for group‐comparisons of thermoregulatory responses (Cramer & Jay, 2014), we opted for a fixed exercise intensity for both the younger and older groups due to its applied relevance (e.g., consider an individual being prescribed some cycling exercise at a gym by a trainer). Nevertheless, by using indirect calorimetry during testing, we were also able to estimate group differences in the rate of heat production during exercise.

Upon termination of the cycling test, participants unmounted the cycle ergometer, dried off with a towel, and their semi‐nude body mass was re‐assessed. This allowed for the calculation of whole‐body sweat loss post‐exercise.

### 2.5 Statistical analysis

#### 2.5.1 Body mapping analysis

First, we evaluated regional differences in local thermal sensation and wetness perception between age groups, by analysing the independent and interactive effects of age (two levels: younger vs. older) and body region (four levels), separately for each stimulus (i.e., cold‐, neutral‐ and warm‐wet), with a two‐way mixed ANOVA. Second, we evaluated generalised differences in wetness perception across the whole body between age groups, by analysing the independent and interactive effects of age (two levels: younger vs. older) and stimulus temperature (three levels: cold‐, neutral‐ and warm‐wet) collapsed over body region (i.e., mean perception of the four regions tested for each participant), with a two‐way mixed ANOVA. Collapsing perceptual data over body regions was deemed relevant to identify the relationship between temperature and wetness (e.g., cold‐wet stimuli induce greater wetness, whereas warm‐wet stimuli suppress the perception of wetness), as recently observed in younger and older males (Wildgoose et al., 2021).

#### 2.5.2 Thermal behaviour analysis

First, we evaluated time‐dependent changes in *H*_prod_ (expressed as W/m^2^), *T*_core_, mean *T*_sk_ and *w* during the 40‐min exercise trial between age groups using a two‐way mixed ANOVA. Data were binned at 60 s and 1‐min averages were used for analysis. Whole‐body sweat loss data were compared between age groups by means of an unpaired Student's *t*‐test.

**FIGURE 2:**
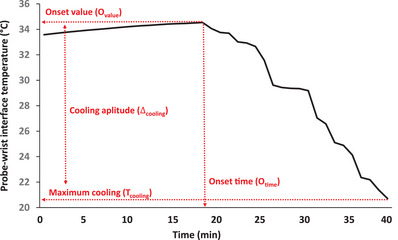
Example of determination of parameters of cool‐seeking behaviour from individual datasets (i.e., in this case from ID2y). Changes in probe–wrist interface temperature were used to determine: (1) onset time (i.e., *O*_time_ corresponded to the time point at which each participant rotated one of the cooling knobs for the first the time from the start of the exercise), and onset value (i.e., *O*_value_ corresponded to the probe‐interface temperature corresponding to the *O*_time_); (2) maximum cooling (i.e., *T*_cooling_ corresponded to the lowest probe–wrist temperature reached during the exercise); and (3) cooling amplitude (Δ_cooling_ corresponded to the difference between the *O*_value_ and *T*_cooling_).

Second, we characterized cool‐seeking behaviour (as indexed by the probe–wrist interface temperature) in terms of its: (1) onset time (i.e., *O*_time_ corresponded to the time point at which each participant rotated one of the cooling knobs for the first time from the start of the exercise), and onset value (i.e., *O*_value_ corresponded to the probe‐interface temperature corresponding to the *O*_time_); (2) maximum cooling (i.e., *T*_cooling_ corresponded to the lowest probe–wrist temperature reached during the exercise); and (3) cooling amplitude (Δ_cooling_ corresponded to the difference between the *O*_value_ and *T*_cooling_). Figure 2 provides a schematic representation of these parameters. We then compared differences in *O*_value_, *O*_time_, *T*_cooling_ and Δ_cooling_ between younger and older women by means of separate unpaired *t*‐tests.

Third, we assessed whether relative changes in *H*_prod_, *T*_core_, mean *T*_sk_ and *w* from the beginning of the exercise to the *O*_time_ differed between age groups by means of separate unpaired *t*‐tests. Furthermore, we used linear regression the establish the association between relative changes in *H*_prod_, *T*_core_, mean *T*_sk_ and *w* from the *O*_time_ until the time point at which *T*_cooling_ was reached and the probe–wrist temperature for each individual participant. We then compared the slopes of the regression lines between age groups by means of separate unpaired *t*‐tests, in order to establish age‐dependent differences in the rate at which changes in *H*_prod_, *T*_core_, mean *T*_sk_ and *w* were associated with changes in probe–wrist temperature during cool‐seeking behaviour.

Fourth, we entered individual data on probe–wrist temperature from the *O*_time_ until the time point at which *T*_cooling_ was reached (i.e., the dependent variable) and the associated changes in *H*_prod_, *T*_core_, mean *T*_sk_ and *w* (i.e., the independent variables) into a multiple linear regression model to identify the relative contribution of each of those biophysical and thermo‐physiological parameters to cool‐seeking behaviours in both younger and older women. All independent variables were log‐transformed to reduce confounding issues associated with multicollinearity in the data set that could affect the resulting β‐coefficients (Slinker et al., 1985). The absolute value of each standardized β‐coefficient for *H*_prod_, *T*_core_, mean *T*_sk_ and *w* from each individual multiple linear regression model was used to calculate the relative contribution of each independent variable for each participant. For example, the relative percentage contribution of a given independent variable (e.g., *H*_prod_) was calculated from the standardized β‐coefficient for this variable (i.e., β (*H*_prod_)) as a function of the sum of all of the standardized β‐coefficients (e.g., β (*H*_prod_)/[β (*H*_prod_) + β (*T*_core_) + β (*T*_sk_) + β (*w*)] × 100). This was done for each variable and for each participant. The relative contributions (%) of each independent variable deriving from the individual standardized β‐coefficients were compared between younger and older women by means of a two‐way mixed ANOVA to establish the extent to which each independent variable explained variance in the cool‐seeking behaviour and any associated age‐dependent difference. This analytical approach was based on that proposed by Vargas et al. (2018a), who recently modelled the relative contribution of core, mean skin temperature and skin wetness to cool‐seeking behaviour during exercise and recovery in young adults.

Finally, we assessed the association between Δ_cooling_ and (1) local cold thermal and wetness sensitivity at the wrist (as determined during the body mapping experiment) and (2) whole‐body warm and cold thermal and wetness sensitivity (i.e., mean of the four tested regions during the body mapping experiment) by means of separate Pearson correlation coefficients.

In the event of statistically significant main effects or interactions, post hoc analyses were conducted with Šidák's test. Normality testing using the Shapiro–Wilk test was performed for all datasets. Multiple linear regression analyses were carried out using SPSS (version 24; IBM Corp., Armonk, NY, USA), while all other analyses were carried out using Prism (version 8.0; GraphPad Software Inc., San Diego, CA, USA). Data are reported as the mean, SD and 95% confidence interval (CI). Observed power was computed using α = 0.05.

## 3 RESULTS

### 3.1 Body mapping of thermal and wetness sensitivity

**FIGURE 3:**
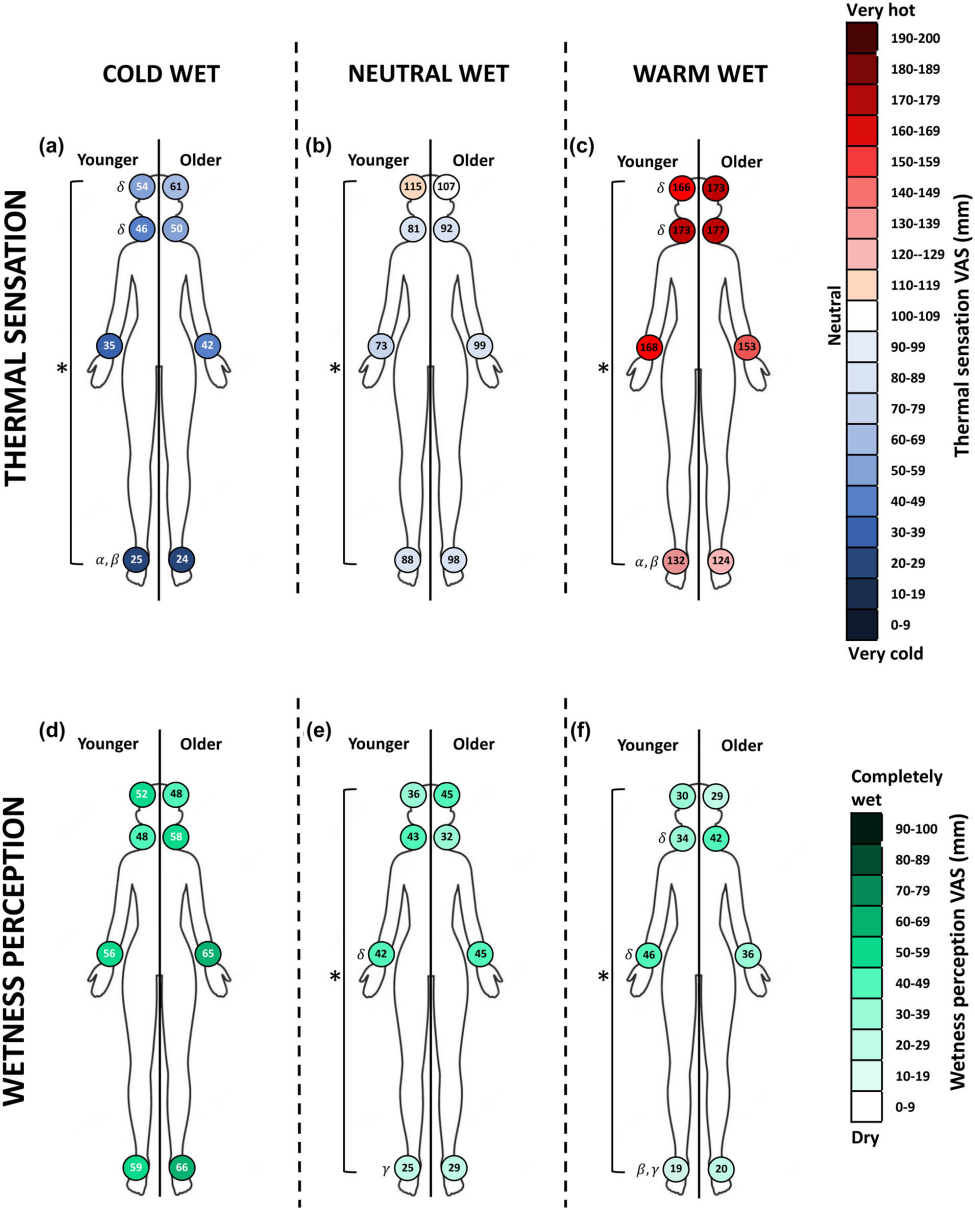
Body maps of thermal sensations and wetness perceptions in younger (*n* = 11) and older women (*n* = 11) resulting from the application of the cold wet (a and d), neutral wet (b and e), and warm wet stimuli (c and f). Numerical data represent group means. Symbols denote statistical differences at *P* < 0.05: ∗, main effect of body region; α, different from forehead; β, different from neck; γ, different from wrist; δ, different from foot. VAS, visual analogue scale.

We found a significant effect of body region, but not age, on thermal sensations (Figure 3) arising from the cold‐wet (body region: *F*_3,60_ = 6.45; *P* = 0.0007; age: *F*_1,20_ = 0.55; *P* = 0.467), neutral‐wet (body region: *F*_3,60_ = 3.16; *P* = 0.031; age: *F*_1,20_ = 2.72; *P* = 0.115) and warm‐wet stimuli (body region: *F*_3,60_ = 10.92; *P* < 0.0001; age: *F*_1,20_ = 0.24; *P* = 0.631). Specifically, cold‐wet stimuli applied to the forehead and the neck were perceived as less cold than when applied to the foot (forehead vs. foot: −32.9 mm; 95% CI: −11.7, −54.1; *P* = 0.0005; corresponding to ∼16% difference; neck vs. foot: −23.2 mm; 95% CI: −2.0, −44.4; *P* = 0.025; corresponding to ∼11% difference). Warm‐wet stimuli applied to the forehead and the neck were perceived as warmer than when applied to the foot (forehead vs. foot: 41.1 mm; 95% CI: 16.8, 65.5; *P* = 0.0001; corresponding to ∼20% difference; neck vs. foot: 46.8 mm; 95% CI: 22.4, 71.1; *P* < 0.0001; corresponding to ∼23% of difference). Of note, we found no differences in warm, neutral or cold sensitivity between the neck and the wrist (warm sensitivity: 14.1 mm; 95% CI: −10.3, 38.4; *P* = 0.541; neutral sensitivity: 0.6 mm; 95% CI: −24.3, 25.6; *P* > 0.999; cold sensitivity: 9.2 mm; 95% CI: −12, 30.5; *P* = 0.809).

When considering wetness perceptions (Figure 3), we found no significant effect of body region or age in response to the cold‐wet stimulus (body region: *F*_3,60_ = 2.329; *P* = 0.083; age: *F*_1,20_ = 0.3542; *P* = 0.558); however, we found a main effect of body region, but not age, in response to the neutral‐wet (body region: *F*_3,60_ = 3.08; *P* = 0.034; age: *F*_1,20_ = 0.04; *P* = 0.846), and warm‐wet stimuli (body region: *F*_3,60_ = 4.55; *P* = 0.006; age: *F*_1,20_ = 0.001; *P* = 0.927). Specifically, greater wetness perceptions were reported at the wrist than the foot in response to the neutral‐wet (wrist vs. foot: 17.2 mm; 95% CI: 0.9, 33.5; *P* = 0.0005; corresponding to ∼17% difference) and warm‐wet stimuli (wrist vs. foot: 21.6 mm; 95% CI: 4.1, 39.1; *P* = 0.008; corresponding to ∼22% difference). Furthermore, greater wetness perceptions were reported at the neck than the foot in response to the warm‐wet stimulus (neck vs. foot: 18.7 mm; 95% CI: 1.2, 36.2; *P* = 0.0300; corresponding to ∼19% of difference). Of note, we found no differences in wetness sensitivity between the neck and the wrist (cold wet: 7 mm; 95% CI: −21.6, 7.6; *P* = 0.729; neutral wet: 6.5 mm; 95% CI: −22.8, 9.8; *P* = 0.863; warm wet: 2.9 mm; 95% CI: −20.4, 14.7; *P* = 0.998).

**FIGURE 4:**
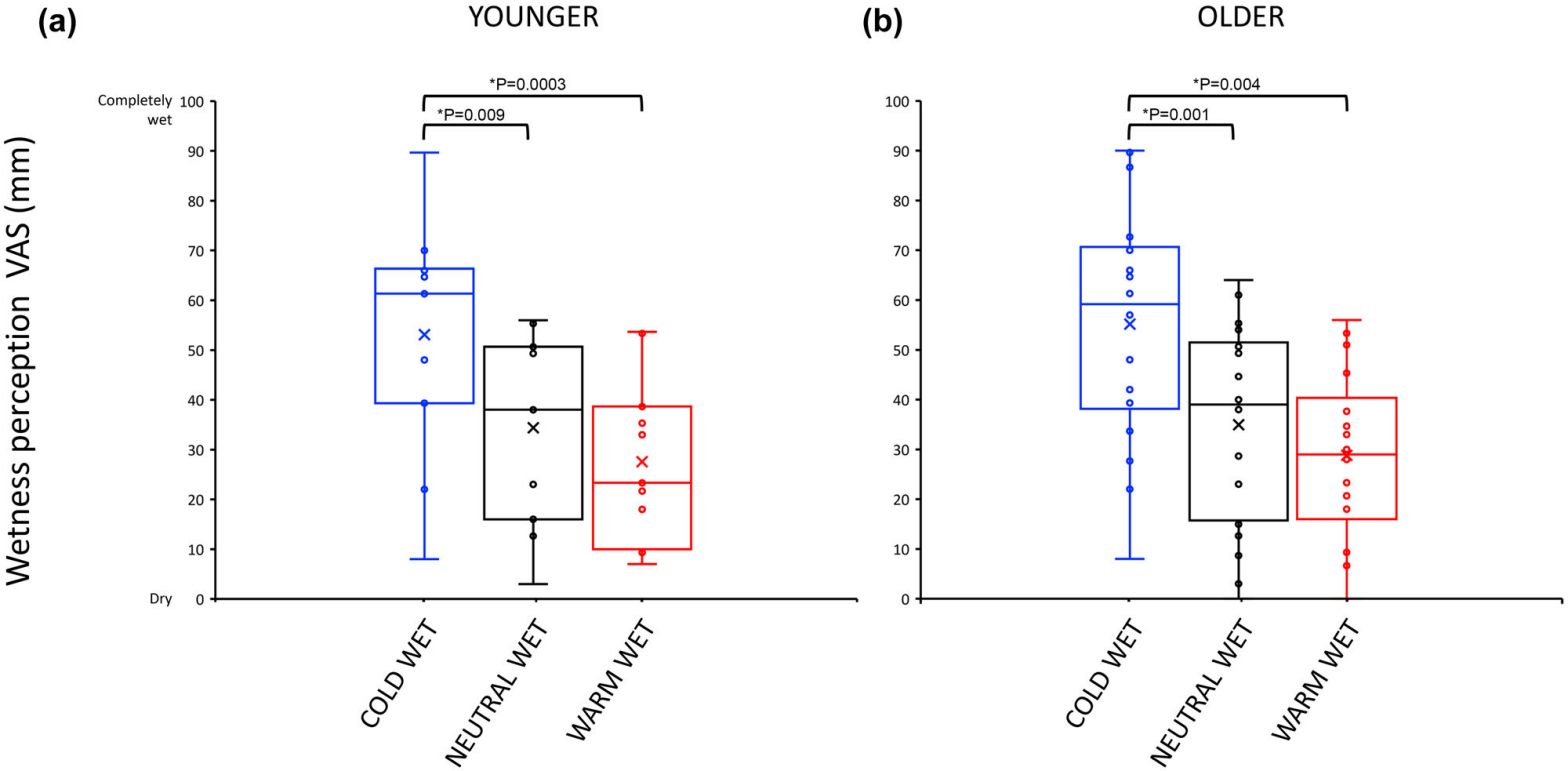
Temperature‐dependent differences in wetness perceptions collapsed over body regions. Box and whisker plots and individual data (*n* = 11 per group) for wetness perceptions arising from the application of the cold‐wet, neutral‐wet and warm‐wet stimuli, in younger (a) and older (b) women. *Main effect of stimulus temperature (*P* < 0.05).

When considering temperature‐dependent differences in wetness perception collapsed over body regions (Figure 4), both younger and older women perceived the cold‐wet stimulus as wetter than the neutral‐wet (difference in the younger group: 17.2 mm; 95% CI: 4.7, 29.6; *P* = 0.009; corresponding to ∼17%; difference in the older group: 21.4 mm; 95% CI: 5.7, 37.0; *P* = 0.001; corresponding to ∼21% difference), and the warm‐wet stimuli (difference in the younger group: 21.5 mm; 95% CI: 12.0, 30.9; *P* = 0.0003; corresponding to ∼22% difference; older: 27.7 mm; 95% CI: 10.4, 45.1; *P* = 0.004; corresponding to ∼28% difference).

### 3.2 Time‐dependent changes in biophysical and thermo‐physiological parameters during exercise

**FIGURE 5:**
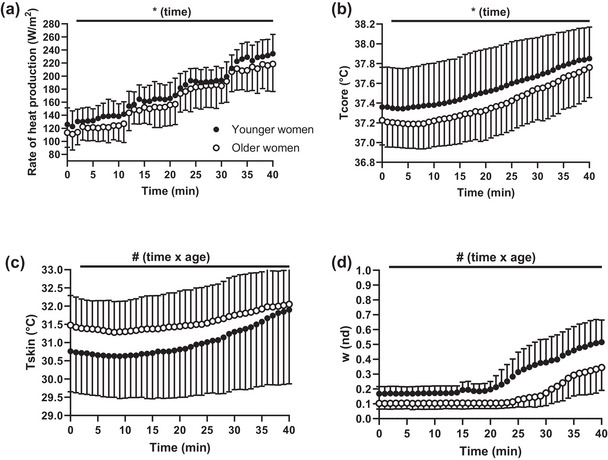
Time‐dependent changes in *H*_prod_ (a), *T*_core_ (b), *T*_sk_ (c), and *w* (d) during the 40‐min incremental cycling test in 11 younger and 11 older women. Data are presented as means (lines) and standard deviations (error bars). *Main effect of time (*P* < 0.05); #interaction time × age group (*P* < 0.05).

Participants’ *H*_prod_ and *T*_core_ increased as a function of time (*F*_39,780_ = 221.3; *P* < 0.0001; *F*_39,780_ = 90.12; *P* < 0.0001, respectively), and they did not differ between age groups (*F*_1,20_ = 1.34; *P* = 0.260; *F*_1,20_ = 1.116; *P* = 0.303, respectively). Specifically, by the end of exercise, *H*_prod_ increased by 108 W/m^2^ (95% CI: 93, 123) in younger women and by 105 W/m^2^ (95% CI: 90, 120) in older women (Figure 5a). By the end of exercise, *T*_core_ increased by 0.49°C (95% CI 0.37, 0.61) in younger women and by 0.53°C (95% CI: 0.41, 0.66) in older women (Figure 5b). Regarding participants’ mean *T*_sk_, we found an interaction between time and age (F_39,780_ = 1.881; *P* = 0.0011), such that the increase in mean *T*_sk_ from the start to the end of exercise was greater in younger (i.e., 1.14°C; 95% CI: 0.75, 1.52) than older women (i.e., 0.57°C; 95% CI: 0.19, 0.95) (Figure 5c). With regards to *w*, we found an interaction between time and age (*F*_39,780_ = 3.816; *P* < 0.0001), such that the increase in *w* from the start to the end of exercise was greater in younger (i.e., 35 dimensionless; 95% CI: 0.25, 0.44) than older women (i.e., 24 dimensionless; 95% CI: 0.15, 0.33) (Figure 5d). Finally, we found that younger women had statistically significantly greater whole‐body sweat losses than their older counterparts (mean difference: 686 g; 95% CI: 6, 1367; *P* = 0.048).

### 3.3 Cool‐seeking behaviour characterization

**FIGURE 6:**
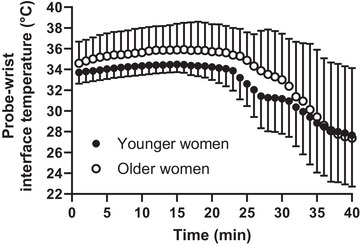
Time‐dependent changes in probe–wrist interface temperature during the 40‐min incremental cycling test in 11 younger and 11 older women. Data are presented as means (lines) and standard deviations (error bars).

Older women had a statistically significant higher *O*_value_ than younger women at the beginning of the cool‐seeking behaviour (mean difference: 1.9°C; 95% CI: 0.3, 3.6; *P* = 0.026) (Figure 6). However, we found no statistically significant difference between younger and older women in the *O*_time_ (mean difference: 1.4 min; 95% CI −4.8, 7.7; *P* = 0.633), Δ_cooling_ (mean difference: 2.2°C; 95% CI: −3.2, 7.7; *P* = 0.406) and *T*_cooling_ (mean difference: −0.3°C; 95% CI: −5.5, 4.9; *P* = 0.908).

When considering the relative changes in *H*_prod_, *T*_core_, mean *T*_sk_ and *w* from the beginning of the exercise to the *O*_time_, as well as the rate of change in these variables during the cool‐seeking behaviour (i.e., the slope of the regression lines), we found no statistically significant differences between age groups in *H*_prod_ (onset value difference: 12.9 W/m^2^; 95% CI: −10.1, 35.8; *P* = 0.257; slope difference: −0.08; 95% CI: −0.18, 0.02; *P* = 0.096), *T*_core_ (onset value difference: 0.04°C; 95% CI: −0.10, 0.18; *P* = 0.519; slope difference: 1.28; 95% CI: −21.33, 23.89; *P* = 0.907) and *w* (onset value difference: −0.04 nd; 95% CI: −0.14, 0.05; *P* = 0.307; slope difference: −3.70; 95% CI: −64.01, 56.6; *P* = 0.899). We also found no differences between age groups in the onset value of mean *T*_sk_ (difference: −0.3°C; 95% CI: −0.5, 0.0; *P* = 0.074), yet we found a statistically significant difference between age groups in the slope of the regression line, such that this was less steep in the older than younger women (slope difference: −12.6; 95% CI: −20.5, −4.6; *P* = 0.004). When considered collectively (i.e., collapsed by age), the mean relative increase in *H*_prod_, *T*_core_, mean *T*_sk_ and *w* from the beginning of the exercise to the *O*_time_ of the cool‐seeking behaviour corresponded to 73.5 ± 26.0 W/m^2^, 0.29 ± 0.15°C, 0.48 ± 0.34°C, and 0.07 ± 0.10 nd, respectively.

### 3.4 Relative contribution of biophysical, thermo‐physiological and perceptual parameters to cool‐seeking behaviour

**FIGURE 7:**
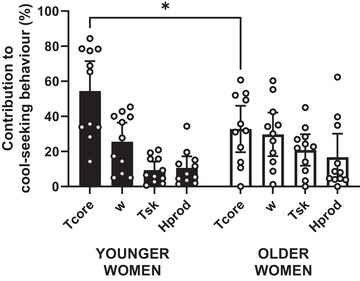
Relative contributions (%) of changes in core temperature (*T*_core_), mean skin temperature (*T*_sk_) and wetness (*w*) and rate of metabolic heat production (*H*_prod_), to participants’ cool‐seeking behaviour in younger and older women. Data are reported as individual data points (dots) and group means and SD (bars). *Statistically significant difference at *P* < 0.05.

The multiple regression models indicated that changes in probe–wrist temperature were primarily described by changes in *T*_core_, followed by *w*, *T*_sk_ and *H*_prod_ in both younger (*R* ^2^ = 0.95 ± 0.05; *P* = 0.010; Table 2) and older women (*R* ^2^ = 0.94 ± 0.07; *P* = 0.032; Table 2) (Figure 7). Specifically, in younger women *T*_core_ explained a significantly greater variance in cool‐seeking behaviour than *w* (mean difference: 28.9%; 95% CI: 5.9, 51.9; *P* = 0.007), *H*_prod_ (mean difference: 43.8%; 95% CI: 20.8, 66.8; *P* < 0.0001), and mean *T*_sk_ (mean difference: 45.1%; 95% CI: 22.1, 68.1; *P* < 0.0001). In older women, *T*_core_ explained a higher, albeit not statistically significant, variance in cool‐seeking behaviour than *w* (mean difference: 3.1% (95% CI: −19.9, 26.1); *P* = 0.999), mean *T*_sk_ (mean difference: 12%; 95% CI: −11, 35; *P* = 0.653), and *H*_prod_ (mean difference: 16.1%; 95% CI: −39.1, 6.9; *P* = 0.319).

**TABLE 2:** Younger and older women's standardized β‐coefficients from linear regressions for the relative contributions of the rate of metabolic heat production (*H*_prod_), core temperature (*T*_core_), mean skin temperature (*T*_sk_), and physical skin wetness (*w*) to cool‐seeking behaviour.

Younger women							Older women						
ID	*H*_prod_	*T*_core_	*T*_sk_	*w*	*R* ^2^	*P*	ID	*H*_prod_	*T*_core_	*T*_sk_	*w*	*R* ^2^	*P*
1y	0.36	1.708	0.965	2	0.924	<0.001	1o	0.044	0.571	0.099	0.385	0.98	<0.001
2y	0.057	1.285	0.011	0.284	0.99	<0.001	2o	0.166	1.139	1.198	1.078	0.978	0.008
3y	0.408	0.17	0.069	0.539	0.964	<0.001	3o	0.63	0.226	0.141	0.012	0.964	<0.001
4y	0.285	0.413	0.229	0.534	0.967	<0.001	4o	0.001	1.098	1.384	0.591	0.789	0.016
5y	0.09	0.565	0.349	0.669	0.9	0.01	5o	0.314	1.933	0.881	0.515	0.966	<0.001
6y	0.186	1.27	0.043	0.12	0.963	<0.001	6o	0.462	0.141	0.375	0.344	0.995	0.001
7y	0.044	1.584	0.036	0.636	1	0.005	7o	0.043	0.152	0.293	0.603	0.988	<0.001
8y	0.017	1.003	0.109	0.06	0.931	<0.001	8o	0.141	0.527	0.035	0.366	0.956	<0.001
9y	0.132	0.792	0.048	0.051	0.993	<0.001	9o	0.512	0	0	0.777	1	0.0
10y	0.225	0.529	0.097	0.636	0.965	0.016	10o	0.134	1.015	0.379	0.145	0.875	<0.001
11y	0.068	0.996	0.239	0.219	0.845	<0.001	11o	0.141	0.964	0.681	1.321	0,929	0.137
Mean	0.170	0.938	0.200	0.523	0.949	0.010	Mean	0.235	0.706	0.497	0.577	0.949	0.032
SD	0.134	0.494	0.275	0.546	0.046	0.006	SD	0.212	0.584	0.477	0.401	0.066	0.059

When comparing the relative contributions of these biophysical and thermo‐physiological parameters to cool‐seeking behaviour between younger and older women, we observed a statistically significant interaction between parameters and age (*F*_(3, 60)_ = 3.05; *P* = 0.035); specifically, we found a statistically significant decrease in the relative contribution of *T*_core_ to changes in probe–wrist temperature in the older women (mean difference with younger women: −21.7%; 95% CI: −3.01, −40.31; *P* = 0.016).

With regard to perceptual parameters, we found no statistically significant correlations between cold thermal and wetness sensitivity at the wrist and Δ_cooling_ in either younger (thermal sensation: *R* ^2^ = 0.1; 95% CI: −0.4, 0.8; *P* = 0.349; wetness perception: *R* ^2^ = 0.02; 95% CI: −0.7, 0.5; *P* = 0.6794) or older women (thermal sensation: *R* ^2^ = 0.08; 95% CI: −0.8, 0.4; *P* = 0.3956; wetness perception: *R* ^2^ = 0.03; 95% CI: −0.7, 0.5; *P* = 0.632). Furthermore, we found no statistically significant correlations between cold thermal and wetness sensitivity averaged across all four tested body regions and Δ_cooling_ in either younger (thermal sensation: *R* ^2^ = 0.06; 95% CI: −0.4, 0.7; *P* = 0.483; wetness perception: *R* ^2^ = 0.02; 95% CI: −0.7, 0.5; *P* = 0.713) or older women (thermal sensation: *R* ^2^ = 0.11; 95% CI: −0.3, 0.8; *P* = 0.317; wetness perception: *R* ^2^ = 0.1; 95% CI: −0.8, 0.4; *P* = 0.354). Finally, we found no statistically significant correlations between warm thermal and wetness sensitivity averaged across all four tested body regions and Δ_cooling_ in either younger (thermal sensation: *R* ^2^ = 0.22; 95% CI: −0.8, 0.2; *P* = 0.149; wetness perception: *R* ^2^ = 0.08; 95% CI: −0.8, 0.4; *P* = 0.399) or older women (thermal sensation: *R* ^2^ = 0.01; 95% CI: −0.7, 0.5; *P* = 0.729; wetness perception: *R* ^2^ = 0.03; 95% CI: −0.5, 0.7; *P* = 0.354).

## 4 DISCUSSION

In relation to our original hypotheses, the results of this study indicated that: (1) older women exhibited a similar cool‐seeking behaviour (i.e., onset and magnitude) to their younger counterparts, despite presenting reductions in their autonomic heat‐dissipation responses (i.e., changes in mean *T*_sk_, physical skin wetness and whole‐body sweat losses) to the same exercise‐induced changes in the rate of metabolic heat production and core temperature; (2) the relative contribution of biophysical, thermo‐physiological, and perceptual parameters to cool‐seeking behaviour changed with ageing, such that older women's thermal behaviour was less heavily determined by changes in core temperature alone (this being a key thermo‐physiological driver in younger women), and more by changes in multiple thermo‐physiological (i.e., *T*_core_, *w* and mean *T*_sk_) and biophysical (i.e., *H*_prod_) parameters; (3) contrary to what we recently observed in aged men (Wildgoose et al., 2021), older women did not present lower regional skin thermal and wetness sensitivity than younger women. We believe that the findings of this study are novel and important, as they provide new insights on female‐specific changes in autonomic and behavioural thermoregulatory responses to exercise performed in a thermoneutral environment across the life course. We consider our experimental approach to this study to be unique in that it combined the evaluation of the relative contribution of biophysical, thermo‐physiological and (importantly) perceptual parameters to cool‐seeking behaviour in women varying in age. As a result, we are in a position to interpret our findings drawing on both the autonomic and perceptual thermoregulatory correlates underlying the observed thermal behaviours. The key implications of our findings are discussed in detail in the section below.

Our first takeaway is that it seems reasonable to conclude that predictions of women's behavioural thermoregulatory responses to exercise performed in a thermoneutral environment should consider the modulatory effect of aging, given that our older women presented both a reduction in their autonomic heat‐defence responses (i.e., primarily whole body sweat loss) and a shift in their reliance from mostly central (i.e., change in *T*_core_) to more integrated central and peripheral thermo‐afferent signals to drive cool‐seeking behaviours.

Regarding autonomic responses, our data fit with those of Kenney & Anderson (1988) and Stapleton et al. (2015), in that older women showed smaller increases in mean *T*_sk_ (indicative of blunted skin vasodilatation) and lower physical skin wetness and whole body sweat losses (indicative of blunted sudomotion), than younger women with similar body mass, surface area, and maximal aerobic capacity, when exercising at the same rate of metabolic heat production in a warm environment (whichever way expressed, i.e., W/m^2^, W/kg, or total *H*_prod_ in W – consider Cramer & Jay (2015). Of note, such blunted heat dissipation responses did not result in disproportionate increases in *T*_core_ in older women. This is not entirely surprising when considering that (a) exercise was performed in thermo‐neutral conditions; (b) reduction in whole‐body sweat loss does not always translate into greater *T*_core_ in both cooler and warmer environments, compared with findings by Allen et al. (2019) and Chaseling et al. (2021).

Perhaps most importantly, despite these autonomic changes, our older women displayed a cool‐seeking behaviour that was as timely and as large (i.e., ∼7°C drop in wrist‐probe temperature) as of that of younger women. Specifically, both age groups experienced a mean relative increase in *H*_prod_, *T*_core_, mean *T*_sk_, and *w* of ∼73 W/m^2^, ∼0.3°C, 0.5°C, and 7% (body surface area covered by sweat at *T*_sk_), respectively, before voluntarily engaging in their cool‐seeking behaviour (whose *O*_time_ was at ∼25 min into the incremental exercise protocol). Our cool‐seeking behaviour paradigm was adapted from that of Vargas et al. (2019a), and hence comparisons can be made on the timing and magnitude of cool‐seeking behaviours across these studies. For example, it is worth noting that, despite undergoing an incremental protocol that resulted in end‐exercise *H*_prod_ levels twice as large as those employed by Vargas et al. (i.e., ∼120 vs. ∼220 W/m^2^), both our and Vargas et al.’s participants achieved a drop in local skin–device temperature of ∼7°C (corresponding to a local *T*_sk_ of ∼27°C) (Vargas et al., 2019a). One may argue that regional differences in thermal sensitivity between the neck region (used by Vargas et al.) and the wrist (used in the current study) may underlie such differential sensitivity (Vargas et al., 2019a); however, our body mapping data indicated that both regions were equally sensitive to cooling, thereby ruling out such a possibility. We therefore cannot exclude that the incremental nature of our protocol (as opposed to Vargas et al.’s steady state approach) (Vargas et al., 2019a), may have resulted in our participants taking a more conservative approach to cooling (i.e., being triggered later on in the protocol). Nevertheless, the fact that an absolute local *T*_sk_ of ∼27°C may be consistently associated with cooling offsetting heat discomfort is intriguing and warrants further investigation.

The second important takeaway of this study is that the relative contribution of biophysical, thermo‐physiological and perceptual parameters to cool‐seeking behaviour changed with ageing, such that older women's thermal behaviour was less heavily determined by changes in core temperature alone (this being a key thermo‐physiological driver in younger women), and more by changes in multiple thermo‐physiological (*T*_core_, *w*, mean *T*_sk_) and biophysical (*H*_prod_) parameters. We speculated that age‐related decreases in the sensitivity of central thermoreceptors innervating the core and viscera may be implicated in this age‐related shift towards more superficial afferent signals (i.e., changes in *w* and mean *T*_sk_) to support cool‐seeking behaviours. This is supported by the fact that older women presented intact skin thermal and wetness sensitivity across their body, unlike the recently reported decline in skin wetness sensitivity observed in older men (Wildgoose et al., 2021). Furthermore, the suggestion that age‐related decreases in the sensitivity of central thermoreceptors innervating the core and viscera may be implicated in changes in both autonomic and behavioural thermoregulation is not entirely unevidenced. For example, such a central mechanism seems to underlie the role of cocaine in blunting both autonomic and perceptual responses to heat stress (Crandall et al., 2002). This hypothesis warrants, of course, further investigation, and we suggest that a relevant model may be one where younger and older males and females are concurrently investigated, as it may provide mechanistic evidence on the extent by which the presence (or absence) of age‐related declines in skin thermo‐wetness sensitivity (such as in the case of men) may differentially impact on the role of central versus peripheral afferents in cool‐seeking behaviour.

While the shift in relative contribution with ageing discussed above is relevant to identify age‐appropriate predictors of heat stress resilience, it is worth noting that the ‘order of importance’ of the biophysical, thermo‐physiological and perceptual parameters involved in cool‐seeking behaviour did not differ between younger and older women. In other words, changes in *T*_core_ still explained the most variance in both groups, followed by *w* and then mean *T*_sk_ and *H*_prod_. Our findings complement the observations of Vargas et al. (2018a), who identified physical skin wetness to be the second most important contributor to cool‐seeking behaviours in the heat, and extend them to include older (female) adults exercising in a thermo‐neutral environment. It therefore appears that, in conjunction with an increase in internal temperature, the build‐up of physical wetness on the skin drives cool‐seeking behaviour to a greater extent than the inputs arising from a rising skin temperature, and that this mechanism is maintained as we age (at least in healthy women).

The third and final take‐away from this study is that, contrary to our initial hypothesis arising from what we recently observed in aged men (Wildgoose et al., 2021), older women did not present lower regional skin thermal and wetness sensitivity than younger women. One potential explanation for these contrasting results may be a slight difference in age between our older women (aged ∼53 years) and Wildgoose et al.’s older men (aged ∼58 years) (Wildgoose et al., 2021), although we consider it unlikely that a ∼5‐year difference may be sufficient to uncover meaningful decreases in somatosensory function between sex groups. Nevertheless, one cannot exclude that age‐dependent decreases in skin wetness sensitivity may be shifted toward older ages in women than men, secondary to women's greater sensitivity to skin wetness, as we previously reported (Valenza et al., 2019). In other words, it may be reasonable to suggest that, as younger women present greater skin wetness sensitivity than men (Valenza et al., 2019), they may also retain this sensory function for longer and experience a decline is sensitivity at older ages than men. The age‐dependency of skin wetness sensitivity across the life course warrants further empirical investigation.

Beside the lack of age‐related differences in skin thermo‐wetness sensitivity, it is worth noting that, as far as we know, this is the first study to have concurrently assessed local skin sensitivity via body mapping and behavioural thermoregulation in the same participant cohort. As a result, we were able to test a commonly reported assumptions in body mapping research (e.g., Gerrett et al.) that individual variability in sensitivities to skin temperature and wetness may be predictive of individual differences in thermal comfort and behaviour under scenarios approaching ecological validity of freely behaving/exercising humans (such as the one adopted in this study) (Gerrett et al., 2014). Somewhat surprisingly, our findings indicated that neither local (wrist) nor whole‐body (i.e., body‐region collapsed) sensitivity to heat, cold and wetness correlated with individual variability in the amplitude of cooling sought during exercise. In other words, we did not find that women who were, for example, more sensitive to cooling at the wrist or less sensitive to heat across their whole body sought less cooling at the wrist to offset their thermal discomfort. It is however important to note that a limitation of this study is that we did not survey whole‐body thermal sensation and discomfort during the exercise trial, as that would have provided a further element of perceptual sensitivity that may have differentiated the resting body mapping sensitivity with participants’ whole‐body sensitivity to discomfort as they experienced exercise‐induced increases in body temperature. Future studies should therefore consider the implications of our findings particularly when developing approaches to the design of wearables (e.g., sport garments) that match skin sensitivities to aid thermal comfort.

### 4.1 Limitations and experimental considerations

There are some experimental considerations to be made when interpreting our findings. First, we did not control for the phase of menstrual cycle of our female participants. There is direct evidence that thermal sensations and exercise performance are not independently modified by menstruation (Matsuda‐Nakamura et al., 2015; McNulty et al., 2020); yet tactile sensitivity (which plays a role in dynamic skin wetness sensitivity) is influenced by the phase of the menstrual cycle (Robinson & Short, 1977). Accordingly, future studies should consider the independent role of menstruation on local skin wetness sensitivity in younger and older women, particularly under dynamic skin interactions with wet stimuli, and its implications on cool‐seeking behaviour particularly during exercise performed in the heat Second, we acknowledge that we also did not control for the role of sex hormones in thermoregulatory responses, which is especially pertinent when considering our older group (i.e., presenting a mix of women either menopausal or being on hormonal replacement therapy). We believe that our diverse sample of older women could be considered a strength when considering the potential application of our findings, for example, to develop cooling wearables that could be widely used by women differing in age and hormonal status; yet the independent effect of hormonal status on thermo‐regulatory and thermo‐behavioural responses remains understudied and warrants further investigation. Third, our experimental protocol involved incremental exercise performed in a thermo‐neutral environment. While our findings are in agreement with those of others who employed either steady‐state protocols (e.g., see Vargas et al., 2019a and the absolute levels of skin cooling achieved by their participants) or exposures to heat stress (e.g., see Kenney & Anderson, 1988 and the blunted thermoregulatory responses in their older participants), readers should therefore be cautious in extending our findings to experimental conditions beyond those tested in this study (i.e., steady‐state exercise performed in a warm environment).

### 4.2 Conclusions

We showed that women's behavioural thermoregulatory responses to exercise are modulated by ageing, such that older women presented both a reduction in their autonomic heat‐defence responses and a shift in their reliance from mostly central to more integrated central and peripheral thermo‐afferent signals to drive cool‐seeking behaviours. Furthermore, and regardless of ageing, neither local nor whole‐body sensitivity to heat, cold and wetness, correlated with individual variability in the amplitude of cooling sought during exercise. These findings have important applied implications to inform the design and development of interventions (e.g., personal cooling systems) and solutions (e.g., body‐mapped sport garments and wearables) that meet the thermal needs of women across different life stages, and that ultimately promote an active life‐style at a time of climate change.

## AUTHOR CONTRIBUTIONS

Alessandro Valenza, Antonino Bianco, Davide Filingeri and Peter R. Worsley conceived and designed the research. Alessandro Valenza and Hannah Blount collected the experimental data. Alessandro Valenza analysed the data and drafted the manuscript. All authors revised the manuscript for intellectual content. All authors have read and approved the final version of this manuscript and agree to be accountable for all aspects of the work in ensuring that questions related to the accuracy or integrity of any part of the work are appropriately investigated and resolved. All persons designated as authors qualify for authorship, and all those who qualify for authorship are listed.

## CONFLICT OF INTEREST

The authors declare that they have no competing interests.

## Data Availability

For the purpose of open access, the author has applied a Creative Commons attribution license (CC BY) to any Author Accepted Manuscript version arising from this submission. Data will be made available upon publication at the University of Southampton data repository (PURE; URL to be activated upon publication).
